# Bridging the distance in precision oncology: a nationwide survey on institutional perspectives toward telemedicine and decentralized trials in Japan

**DOI:** 10.1007/s10147-026-03034-x

**Published:** 2026-04-30

**Authors:** Hiroya Taniguchi, Takeshi Kawakami, Hironaga Satake, Yuko Tamura, Takuma Matsunaga, Yu Sunakawa

**Affiliations:** 1 Department of Clinical Oncology. Aichi Cancer Center Hospital, 1-1 Kanokoden, Chikusa-ku, Nagoya, Aichi 464-8681 Japan; 2 Division of Gastrointestinal Oncology, Shizuoka Cancer Center, Shizuoka, Japan; 3 Department of Medical Oncology, Kochi Medical School, Kochi, Japan; 4 NTT DOCOMO BUSINESS, Inc., Tokyo, Japan; 5 MICIN, Inc., Tokyo, Japan; 6 Department of Clinical Oncology, St. Marianna University School of Medicine, Kanagawa, Japan

**Keywords:** Genomic profiling, Telemedicine, Decentralized clinical trials, Access to care, Precision oncology

## Abstract

**Background:**

Comprehensive genomic profiling (CGP) has been reimbursed under Japan’s universal health coverage since 2019, yet access remains uneven because designated institutions are concentrated in urban areas. Although CGP can identify actionable alterations, fewer than 10% of patients receive matched therapies, often due to geographic barriers to clinical trial enrollment. This nationwide survey evaluated institutional challenges in CGP implementation and explored the perceived role of telemedicine and decentralized clinical trials (DCTs) in improving equitable access.

**Methods:**

A web-based questionnaire was distributed to professionals involved in CGP across government-designated hospitals for cancer genomic medicine between December 2024 and February 2025. Respondents provided institutional data and perceptions regarding CGP access, telemedicine use, and barriers to trial participation.

**Results:**

A total of 194 professionals from 140 hospitals responded. On average, 23.5% of CGP-tested patients were referred from non-designated hospitals, often requiring multiple in-person visits. Fifty-eight percent of respondents supported telemedicine for both informed consent and result disclosure, citing reduced travel burden, while concerns included digital literacy and institutional workload. Sixty-one percent reported that over half of eligible patients declined genotype-matched trials due to travel distance. Institutions facing higher dropout rates expressed greater support for DCTs (67% “strongly needed”).

**Conclusion:**

This nationwide study identified major geographic and logistical barriers to equitable precision oncology in Japan. Most institutions view telemedicine and DCTs as essential to expanding access to genomic testing and clinical trials. Japan’s experience may offer insights for other healthcare systems considering integration of telemedicine into national precision oncology frameworks under universal health coverage.

**Supplementary Information:**

The online version contains supplementary material available at 10.1007/s10147-026-03034-x.

## Introduction

The emergence of next-generation sequencing and the wider availability of genomically targeted therapies have accelerated the global adoption of comprehensive genomic profiling (CGP) in clinical care. Japan is among the first countries to integrate CGP into a nationwide, reimbursed cancer care program under its universal health coverage system, established in 2019 [1, 2]. Despite this system, access remains uneven because CGP testing is limited to ~ 280 government-designated hospitals equipped with molecular tumor boards, genetic counseling, and clinical trial infrastructure. Patients receiving care in non-designated or rural hospitals therefore often face restricted access, and insurance coverage generally applies only after standard therapies, further limiting uptake [3].

Even when CGP identifies actionable genomic alterations, fewer than 10% of patients ultimately receive matched therapies [4–7]. This low implementation rate is partly due to limited enrollment in genotype-directed clinical trials. Although CGP testing may be available at a designated facility, patients can still lack access to matched treatments if relevant clinical trials are held at distant locations. Such trials are largely conducted in a small number of high-volume cancer centers in metropolitan areas. Therefore, patients in rural or underserved regions face substantial logistical and financial barriers, including long-distance travel, transportation and accommodation costs, and time away from home, with expenses rarely being reimbursed.

Telemedicine has rapidly expanded worldwide [8], including in oncology, where it has improved access and patient satisfaction, its adoption in Japan remains limited. For CGP testing, informed consent and results disclosure via telemedicine are not reimbursed, and during the coronavirus disease 2019 (COVID-19) pandemic, remote consultations were largely confined to exceptional cases. As a result, patients referred from nondesignated facilities must visit CGP centers multiple times, compounding the physical and psychological burdens of cancer care. Similarly, clinical trial participation has traditionally required in-person site visits, further limiting access [9]. Although pilot efforts have tested decentralized clinical trial (DCT) models using telemedicine, where local diagnostic procedures are paired with remote trial oversight [10], such practices remain rare, and most genotype-matched trials continue to depend on face-to-face protocols.

To better understand the aforementioned challenges, we conducted a nationwide survey of CGP-designated institutions to assess perceived barriers and institutional needs related to CGP use and clinical trial participation. In parallel, we examined the potential role of telemedicine in expanding precision oncology access. This study marks an early effort to identify systemic obstacles and inform strategies to promote equitable genomic medicine access across Japan.

## Patients and methods

### Participants

We conducted a web-based survey of healthcare professionals involved in CGP in Japan. Eligible participants included physicians, nurses, and cancer genome coordinators engaged in CGP-related care at one of the designated CGP hospitals, classified into Core, Designated, Cooperative Hospitals for Cancer Genomic Medicine—the official categories defined by Japan’s Ministry of Health, Labour and Welfare. Participants were required to be employed in a CGP Hospital. Completion of the survey was regarded as providing informed consent to participate. The survey was conducted during December 2024–February 2025. Hospitals were classified as either urban or rural based on geographic location. Urban hospitals were defined as those located in the Tokyo, Osaka, Nagoya, Fukuoka, or Sapporo metropolitan areas. All other hospitals were categorized as rural. Although formal weighting was not applied, the distribution of responding institutions across designation categories and regions appeared broadly consistent with national figures.

### Data collection

The survey was distributed via email using contact networks affiliated with CGP implementation sites. The email included an invitation and a link to the online questionnaire. Respondents answered initial screening questions to confirm eligibility. Each institution was asked to provide responses from one to three individuals, including at least one physician. For selected items, such as the estimated number of CGP tests conducted annually and the number of patients referred from nondesignated institutions, respondents were allowed to provide approximate figures based on institutional records or informed estimates. These data were not independently verified against registry or audited datasets.

### Data analysis

Descriptive statistics, including frequencies and proportions, were calculated using EZR (Easy R), a graphical user interface for R (The R Foundation for Statistical Computing), to analyze quantitative data. The proportion of missing responses was calculated for each item and reported in the results. Free-text responses were reviewed and summarized narratively by the research team. No inferential statistical tests were conducted, as the study’s primary objective was to explore general trends and institutional perceptions rather than test specific hypotheses.

## Results

### Participant characteristics

From a total of 290 designated hospitals, 194 healthcare professionals from 140 institutions (48%) responded to the survey. These included 140 physicians and 54 nonphysician staff, such as nurses and cancer genome coordinators. Of all respondents, 37% were affiliated with urban institutions and 63% with rural institutions. Participating institutions comprised 10 Designated Core Hospitals for Cancer Genomic Medicine, 10 Designated Hospitals, and 120 Cooperative Hospitals across Japan.

**Fig. 1:**
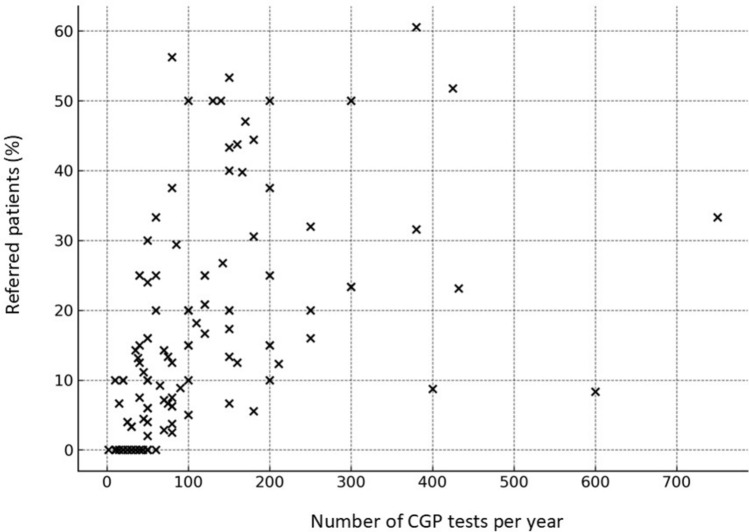
Annual number of comprehensive genomic profiling (CGP) tests performed at each institution (x-axis) and the corresponding proportion of patients referred from nondesignated hospitals (y-axis). Each point represents a single institution

Collectively, these 140 institutions conducted 15,041 CGP tests per year. Among these, 3542 patients (23.5%) were referred specifically for CGP from non-designated institutions not authorized to perform such testing. However, the referral rate varied widely among institutions. Notably, there was considerable variability in the proportion of referred patients relative to total CGP tests performed at each institution (Fig. 1). Some low-volume facilities reported high referral rates, and even among high-volume centers, referral proportions varied markedly. Moreover, the variability in referral rates did not significantly differ between urban and rural hospitals (data not shown). Patients referred from non-designated institutions typically need to visit CGP centers twice—once for informed consent and sample collection, and again for results disclosure—each requiring long-distance travel. This process often imposes substantial time and financial burdens, particularly for patients living in rural areas.

### Perceptions of telemedicine use in CGP testing

**Fig. 2:**
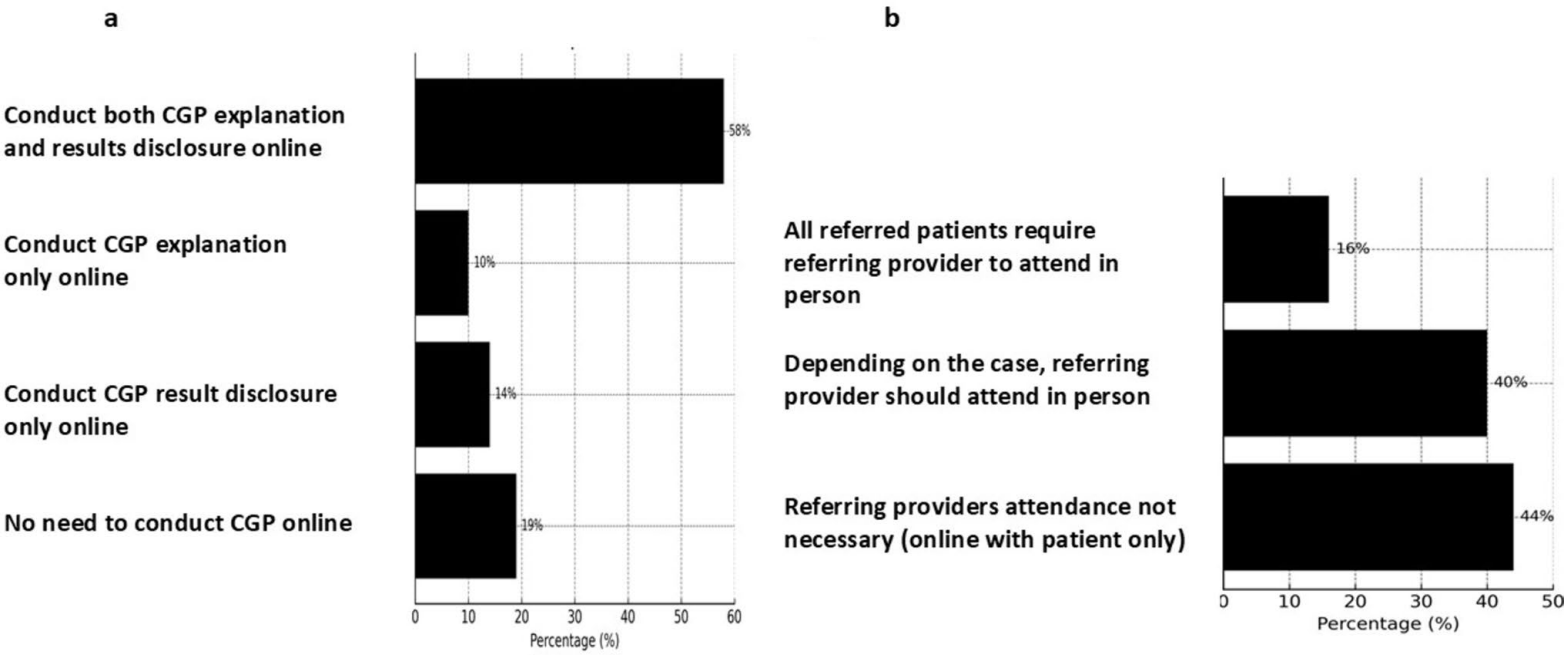
**a** Responses to the question: “Do you think online consultation is needed in CGP testing, such as for informed consent and results explanation, to reduce patient burden, especially for those referred from nongenomic centers?” **b** Responses to the question: “When conducting online CGP consultations (e.g., for informed consent or results disclosure) for patients referred from nongenomic hospitals, is it necessary for medical personnel from the referring institution to attend the session?”

When asked about the potential role of telemedicine in CGP testing, particularly for reducing travel burden among patients referred from non-designated institutions, 58% of respondents supported using telemedicine for informed consent and results disclosure. An additional 10% favored using it for informed consent only, whereas 14% supported online results explanation alone. In contrast, 19% of respondents felt that CGP-related procedures should not be conducted online (Fig. 2a).

### Requirement for the referring healthcare provider’s presence during telemedicine

Respondents were also asked whether referring healthcare providers (e.g., primary physicians or nurses from non-designated institutions) should be present during CGP-related telemedicine sessions. Although 16% indicated that such providers should always be present, 40% believed that their presence should depend on the case. The remaining 44% considered their presence unnecessary, stating that telemedicine involving only the patient and family would suffice in most cases (Fig. 2b).

### Free-text responses on expectations for online CGP implementation

Free-text comments emphasized that telemedicine is critical for equitable access to genomic medicine, especially for patients in rural areas or with limited transportation (Table S1). Many noted that CGP-related consultations rarely require repeat visits, making them well suited for remote delivery and reducing physical, emotional, and financial burdens associated with long-distance travel. Several clinicians described positive experiences with telemedicine during the COVID-19 pandemic or when patients were too ill to visit the hospital, noting that explanations and follow-up care were largely equivalent to in-person encounters because facial expressions and vocal tone remain visible online. Respondents also felt that online systems could mitigate regional disparities, especially in areas lacking clinicians trained in CGP management. Some highlighted that limited CGP access has become a bottleneck for downstream treatment, including clinical trial enrollment, and viewed telemedicine as a promising tool to facilitate earlier engagement with trial sites—particularly for younger, highly motivated patients.

### Free-text responses regarding barriers and considerations for online implementation of CGP

Several barriers to implementing telemedicine for CGP testing were identified (Table S2). In-person visits were often preferred, especially when disclosing hereditary cancer results or involving genetic counselors. Concerns included the separation of results disclosure and counseling potentially reducing follow-up completion as well as the complexity of CGP testing making online consent challenging. Evaluating patient eligibility, such as performance status, was viewed as difficult without face-to-face interaction, and it was thought that elderly patients would struggle with digital tool use. Variability in CGP literacy across institutions further complicated remote communication.

Logistical issues were also highlighted, including infrastructure needs, unclear institutional benefits, and concerns that telemedicine could increase workload without improving efficiency. Privacy risks, such as data confidentiality and unauthorized recordings, were also noted. Finally, effective coordination with referring physicians was considered time-consuming, and many respondents reported that in-person referrals remain the prevailing practice.

### Travel-related barriers to clinical trial participation

**Fig. 3:**
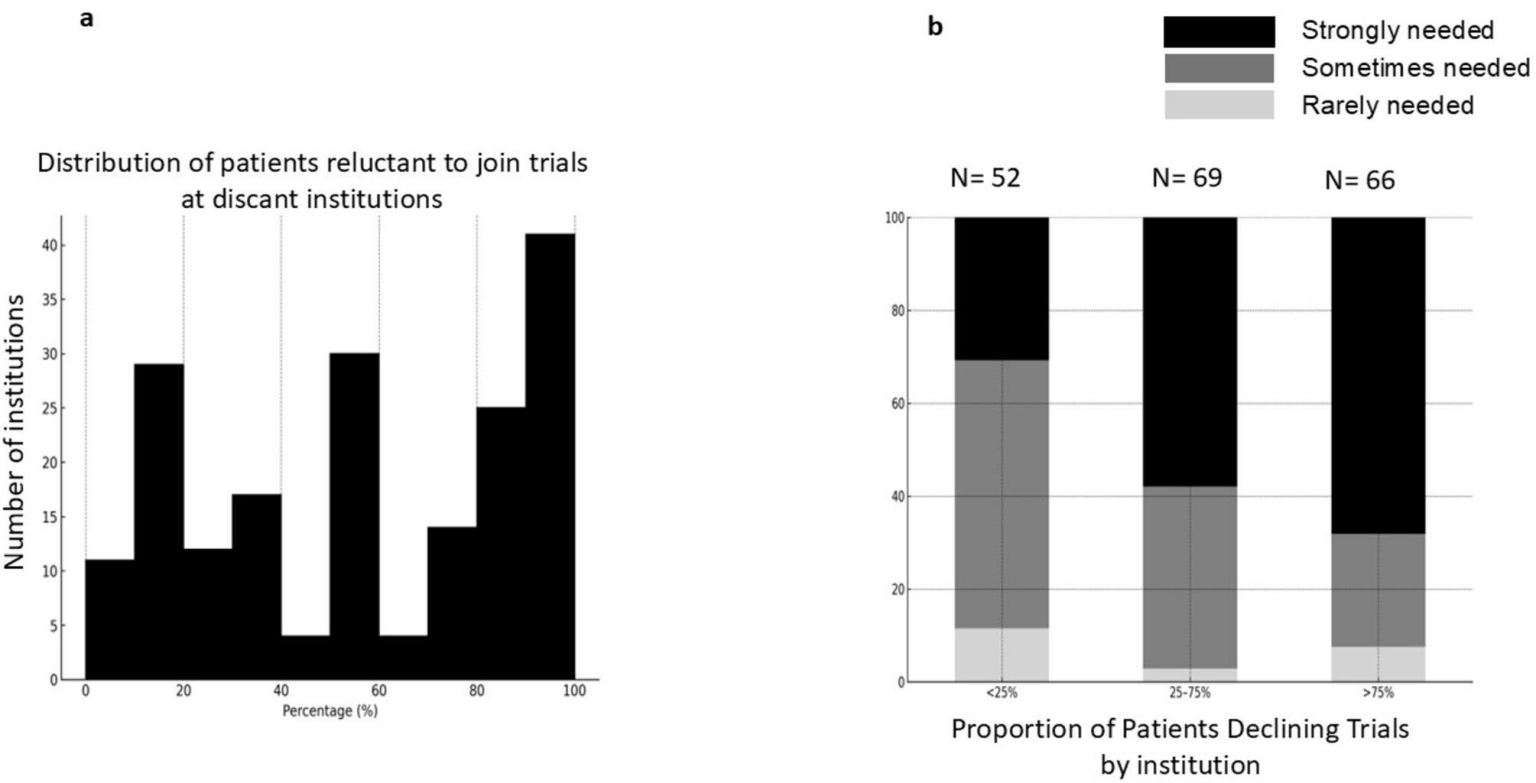
Distribution of institutional estimates regarding the percentage of patients who decline or are not referred to genotype-matched clinical trials owing to the distant location of trial sites. Each bar represents the number of institutions reporting within a specified percentage range (e.g., 0–10%, 10–20%, etc.). **b** Perceived need for decentralized clinical trials (DCTs) stratified by the estimated proportion of patients declining participation due to travel burden. Institutions reporting higher dropout rates more frequently considered DCTs, incorporating telemedicine and local test administration, to be “very necessary.”

Participants estimated the proportion of patients who, despite identification of actionable genomic alterations through CGP, ultimately declined or were not referred to clinical trials owing to the trial site’s distant location. Responses varied (Fig. 3a), but the trend highlighted a major geographic barrier: 61% of respondents reported that > 50% of eligible patients declined trials because of travel burden. Notably, 35% indicated that > 80% of such patients abandoned trial opportunities, suggesting that long-distance travel is a major deterrent, even when matched therapies are available. The distribution of trial abandonment rates differed significantly by region (p < 0.001). Notably, institutions outside major metropolitan areas (Tokyo, Osaka, Nagoya, Fukuoka, and Sapporo) reported a substantially higher proportion of patients declining participation in clinical trials due to geographic barriers (data not shown).

In a related question, respondents were asked to specify the travel time beyond which they would hesitate to refer patients to a clinical trial site (assuming biweekly visits). The most common threshold was “ ≥ 2 h,” selected by 97 responders, with another 35 indicating “ ≥ 3 h” as a critical barrier. Only 14 responders stated that travel time or distance did not influence their referral decisions (data not shown).

### Perceived need for DCTs using telemedicine

To evaluate interest in DCTs, respondents were asked whether trial sponsors should offer remote online participation models while allowing laboratory tests or drug administration at local sites. Overall, 54% stated that such trials are “strongly needed,” with an additional 39% answering that they are “sometimes needed,” and only 7% indicating that they are "rarely needed."

A stratified analysis based on the proportion of patients who declined trial participation owing to travel distance revealed a clear trend: institutions with higher abandonment rates reported a stronger need for online-enabled trials. Specifically, among institutions where > 75% of eligible patients declined participation because of a travel burden, 67% considered such trials “strongly needed,” compared to 29% among institutions with < 25% dropout. Conversely, the proportion of respondents indicating that the trials were “rarely needed” decreased as dropout rates increased (Fig. 3b). These findings suggest that support for DCTs correlates with the real-world barriers patients face in reaching centralized trial sites.

### Concerns regarding DCT implementation

**Fig. 4:**
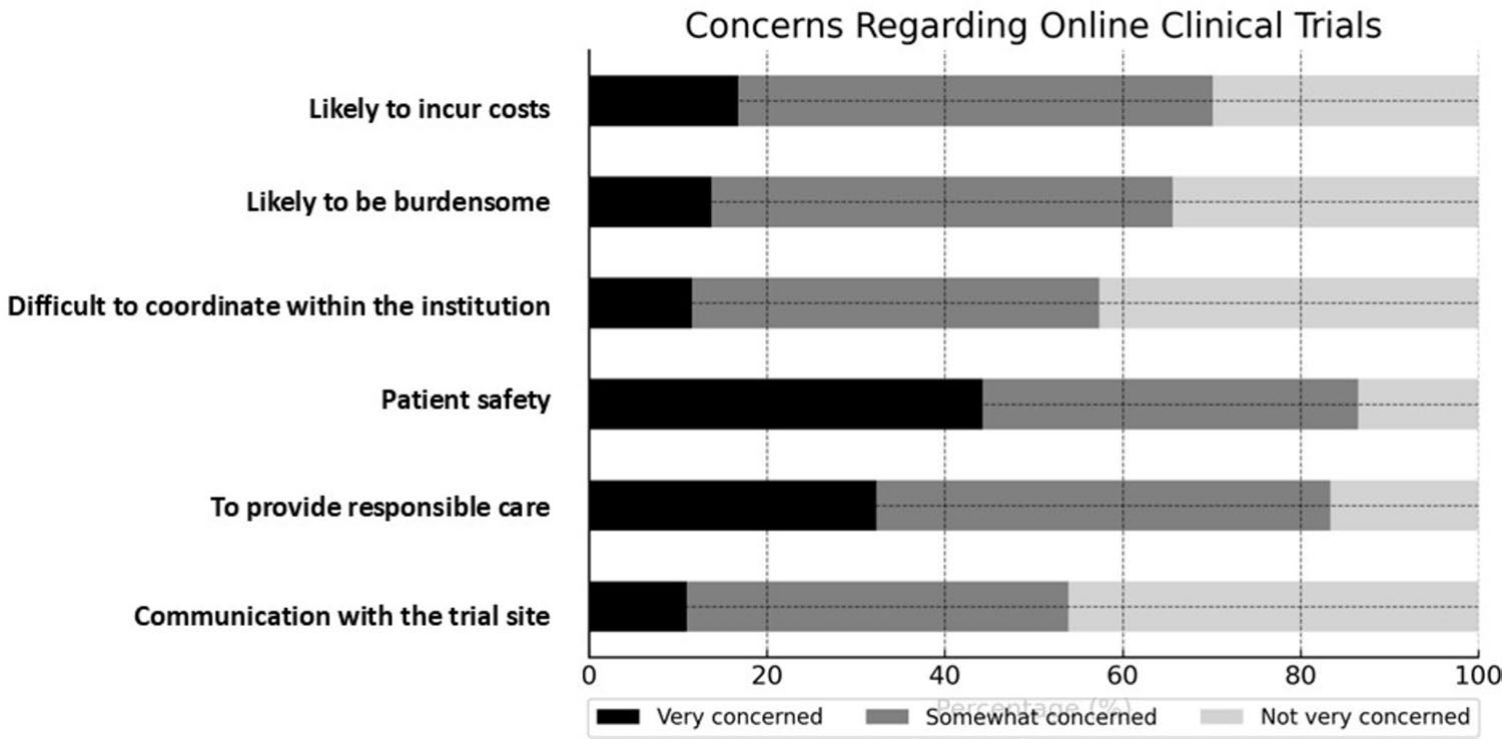
Respondent concerns regarding implementing decentralized clinical trials, categorized by domain (e.g., cost, coordination, and patient safety). Responses are grouped by level of concern: "Very concerned," "Somewhat concerned," and "Not very concerned"

Respondents were also asked about concerns related to implementing DCTs. The most common issues were internal coordination difficulties (reported by > 80%) and a projected increase in workload (Fig. 4). These results indicate that, beyond clinical or ethical concerns, operational readiness remains a major hurdle to wider adoption. Additional issues included patient safety, the ability to provide responsible care remotely, and communication with the central trial site. Financial costs and system reliability were also cited, although less frequently.

## Discussion

This nationwide survey highlights structural barriers and emerging opportunities in improving equitable access to precision oncology in Japan. Although CGP testing has been formally integrated into the national health system, access remains geographically limited, with many patients referred from non-designated institutions and requiring multiple in-person visits to specialized centers. As our findings indicate, these logistical burdens create substantial disparities, especially for patients in rural areas.

Telemedicine for CGP-related consultations is not reimbursed in Japan; however, 58% of respondents supported conducting both steps remotely, with another 24% endorsing partial online use. Respondents noted that remote consultations could ease physical, emotional, and financial strain, particularly for patients in rural areas facing long travel times. Given that CGP discussions are typically infrequent and primarily informational, they are well-suited for telemedicine. Internationally, virtual molecular tumor boards have been used to support genomic interpretation and treatment decisions across institutions [11, 12]. These platforms enable clinicians at non-designated facilities to access expert guidance and deliver timely, genomically informed care.

Prior studies support the potential of telemedicine in this context. For instance, one study found that 57% of patients with cancer preferred telemedicine for providing informed consent, given its convenience and the reduced travel burden [13]. Moreover, a systematic review and meta-analysis of randomized trials showed that videoconferencing-based telemedicine in oncology outpatient care yields comparable outcomes to in-person visits in terms of patient satisfaction, appointment adherence, consultation duration, and psychological well-being [14]. Even for results disclosure, which often involves communicating sensitive information, telemedicine has proven effective, with no significant difference reported in patient distress when negative news was delivered remotely [15]. These data affirm the feasibility of using telemedicine for CGP-related consultations. In Japan, remote genetic counseling is already in place at several institutions. These precedents offer a practical foundation for expanding CGP-related telemedicine practices. Broader adoption could improve equity and access to genomic medicine. To realize this, regulatory and reimbursement frameworks must evolve in parallel with efforts to enhance data security, provider preparedness, and digital access for underserved groups.

Nevertheless, feasibility may vary across patient populations. For elderly patients or those with limited digital literacy, hybrid models combining local nurse-assisted teleconsultation or caregiver-supported participation may improve feasibility. Structured digital navigation support and simplified consent interfaces could further enhance accessibility. Additionally, stratified approaches—reserving in-person visits for clinically complex cases while offering telemedicine for stable or informational encounters—may optimize both safety and efficiency.

One major reason for the low treatment implementation rate, reported as < 10% even when actionable genomic alterations are identified via CGP, is limited clinical trial access due to geography. Our survey revealed that 61% of institutions estimated that > 50% of eligible patients declined trial participation owing to their travel burden. Many respondents were hesitant to refer patients to trials requiring > 2 h of travel. This finding aligns with previous studies demonstrating an inverse relationship between travel distance and trial enrollment [16, 17]. The perceived need for DCTs was particularly high among institutions with elevated patient dropout rates due to distance, with 54% and 39% stating that DCTs are “strongly needed” and “somewhat needed,” respectively. These results underscore the potential of DCTs to reduce disparities and broaden trial access.

Within Japan’s healthcare infrastructure, this challenge is especially striking. Many regional general hospitals are equipped with advanced imaging and laboratory capabilities, including computed tomography, magnetic resonance imaging, and positron emission tomography–computed tomography, which are often sufficient to support trial-related assessments [18]. This environment provides a strong foundation for DCT model implementation, in which patients undergo routine procedures locally while connecting with central trial sites through telemedicine [19]. In recent years, regulatory changes in Japan have further supported DCT expansion. New guidelines on electronic informed consent and remote data collection, introduced in 2023 and 2024, respectively, mark key steps toward operationalizing online-enabled trials. Pilot initiatives, such as satellite site collaborations, have begun to demonstrate feasibility and scalability in oncology [10], although challenges remain. Respondents cited institutional coordination, workload increases, data privacy, and unclear responsibilities for patient safety as key concerns. These findings highlight the need for practical implementation frameworks, including reimbursement schemes, training for providers, and digital literacy support for patients.

This study has several limitations. Although responses were obtained from nearly half of all CGP-designated hospitals nationwide, selection bias cannot be excluded. Institutions with greater interest in telemedicine or stronger engagement in CGP may have been more likely to participate. Therefore, the findings may overrepresent institutions already motivated toward digital transformation, potentially limiting generalizability. Moreover, several quantitative measures were based on self-reported institutional estimates rather than audited registry data. Although respondents were encouraged to refer to institutional records, misclassification or recall bias may have affected numerical accuracy.. Additionally, our analysis of barriers to clinical trial participation did not account for patients who may have been ineligible owing to advanced age, poor performance status, or other clinical factors. The study is also subject to typical survey limitations, including response bias, interpretative variability, and inconsistent institutional practices. Importantly, this survey reflects institutional and healthcare professional perspectives and does not directly capture patient-reported experiences, preferences, or perceived burdens. Future research incorporating patient-reported outcomes and qualitative interviews will be essential to validate whether institutional perceptions align with patient needs and to design patient-centered telemedicine and DCT frameworks. Finally, Japan’s centralized CGP designation system and universal health coverage structure represent a unique healthcare environment. In countries with more decentralized or insurance-based systems, logistical, regulatory, and reimbursement barriers may differ substantially. Therefore, extrapolation of these findings should be undertaken cautiously, taking into account country-specific infrastructure and policy contexts.

In summary, our nationwide survey highlighted major barriers to equitable CGP and genotype-matched clinical trial access in Japan, especially due to geographic and institutional limitations. Our findings emphasize the strong clinical support for telemedicine and DCT models to reduce patient burden and expand participation. With aligned policy reform, infrastructure development, and operational support, broader adoption of remote precision oncology could close access gaps and expand treatment options for patients across diverse regions.

## Supplementary Information

Below is the link to the electronic supplementary material.

Supplementary file1 (PDF 51 kb)

## Data Availability

The datasets generated and/or analyzed during the current study are not publicly available due to ethical and patient privacy restrictions. De-identified data may be made available from the corresponding author on reasonable request.

## References

[1] Ebi H, Bando H (2019) Precision oncology and the universal health coverage system in Japan. JCO Precis Oncol 3:1–12. 10.1200/PO.19.00291 32923862 10.1200/PO.19.00291 PMC7446489

[2] Ando Y, Shimoi T, Suzuki T et al (2023) Genomic medicine in clinical practice: national genomic medicine program in Japan. Cancer Biol Med 21(1):4–9. 10.20892/j.issn.2095-3941.2023.0219 37818596 10.20892/j.issn.2095-3941.2023.0219 PMC10875283

[3] Hagio K, Kikuchi J, Takada K et al (2023) Assessment for the timing of comprehensive genomic profiling tests in patients with advanced solid cancers. Cancer Sci 114(8):3385–3395. 10.1111/cas.15837 37208840 10.1111/cas.15837 PMC10394138

[4] Sunami K, Naito Y, Komine K et al (2022) Chronological improvement in precision oncology implementation in Japan. Cancer Sci 113(11):3995–4000. 10.1111/cas.15517 35976133 10.1111/cas.15517 PMC9633287

[5] Hayashi N, Mori S, Okamoto A et al (2024) Availability of genome-matched therapy based on clinical practice. Int J Clin Oncol 29(7):964–971. 10.1007/s10147-024-02533-z 38668785 10.1007/s10147-024-02533-z PMC11196305

[6] Meric-Bernstam F, Brusco L, Shaw K et al (2015) Feasibility of large-scale genomic testing to facilitate enrollment onto genomically matched clinical trials. J Clin Oncol 33(25):2753–2762. 10.1200/JCO.2014.60.4165 26014291 10.1200/JCO.2014.60.4165 PMC4550690

[7] Zehir A, Benayed R, Shah RH et al (2017) Mutational landscape of metastatic cancer revealed from prospective clinical sequencing of 10,000 patients. Nat Med 23(6):703–713. 10.1038/nm.4333 28481359 10.1038/nm.4333 PMC5461196

[8] Lopez AM (2024) Telehealth in cancer care: inequities, barriers, and opportunities. Cancer J 30(1):2–7. 10.1097/PPO.0000000000000694 38265919 10.1097/PPO.0000000000000694 PMC10904017

[9] Takamizawa S, Koyama T, Sunami K et al (2024) Identification of barriers to implementation of precision oncology in patients with rare cancers. Cancer Sci 115(6):2023–2035. 10.1111/cas.16165 38538548 10.1111/cas.16165 PMC11145155

[10] Taniguchi H, Masuishi T, Ogata T et al (2023) First experience of a fully decentralized clinical trial: the dawn of a new era in oncology. Cancer Sci 114(7):3050–3052. 10.1111/cas.15792 36971104 10.1111/cas.15792 PMC10323108

[11] Angel M, Demiray M, Disel U et al (2024) The value of virtual molecular tumor boards for informed clinical decision-making. Oncologist 29(7):554–559 38761380 10.1093/oncolo/oyae077 PMC11224979

[12] Pishvaian M, Blais E, Bender RJ et al (2019) A virtual molecular tumor board to improve efficiency and scalability of delivering precision oncology to physicians and their patients. JAMIA Open 2(4):505–515. 10.1093/jamiaopen/ooz045 32025647 10.1093/jamiaopen/ooz045 PMC6994017

[13] Khadem Charvadeh Y, Doshi SD, Seier K et al (2025) Cancer patient perspectives on clinical trial discussion and informed consent through telemedicine. JCO Oncol Pract 21(10):1439–1446. 10.1200/OP-24-00764 40101176 10.1200/OP-24-00764 PMC12353420

[14] Uemoto Y, Yamanaka T, Kataoka Y et al (2022) Efficacy of telemedicine using videoconferencing systems in outpatient care for patients with cancer: a systematic review and meta-analysis. JCO Clin Cancer Inform 6:e2200084. 10.1200/CCI.22.00084 36417685 10.1200/CCI.22.00084

[15] Mueller J, Beck K, Loretz N et al (2023) The disclosure of bad news over the phone vs. in person and its association with psychological distress: a systematic review and meta-analysis. J Gen Intern Med 38(16):3589–3603. 10.1007/s11606-023-08323-z 37552418 10.1007/s11606-023-08323-z PMC10713955

[16] Meropol NJ, Buzaglo JS, Millard J et al (2007) Barriers to clinical trial participation as perceived by oncologists and patients. J Natl Compr Canc Netw 5(8):655–664. 10.6004/jnccn.2007.0067 17927923 10.6004/jnccn.2007.0067

[17] Uehara Y, Koyama T, Katsuya Y et al (2023) Travel time and distance and participation in precision oncology trials at the National Cancer Center Hospital. JAMA Netw Open 6(9):e2333188. 10.1001/jamanetworkopen.2023.33188 37713200 10.1001/jamanetworkopen.2023.33188 PMC10504617

[18] Aoyama T, Koide Y, Shimizu H et al (2025) A cross-national investigation of CT, MRI, PET, mammography, and radiation therapy resources and utilization. Jpn J Radiol 43(1):109–116. 10.1007/s11604-024-01650-z 39240460 10.1007/s11604-024-01650-z

[19] Fu S, Gerber D, Beg MS (2023) Decentralized clinical trials in oncology: are we ready for a virtual-first paradigm? J Clin Oncol 41(2):181–185. 10.1200/JCO.22.00358 35994691 10.1200/JCO.22.00358 PMC9839231

