# Supplementary material for: Bridging the distance in precision oncology: a nationwide survey on institutional perspectives toward telemedicine and decentralized trials in Japan
Source: Int J Clin Oncol. 2026 Apr 30;31(7):1275–82. doi: 10.1007/s10147-026-03034-x (PMC13303474; doi:10.1007/s10147-026-03034-x)
Supplement: Supplementary file 1 — Supplementary file1 (PDF 51 kb) [file 10147_2026_3034_MOESM1_ESM.pdf]

Table S1 Expectations for Online Implementation of CGP

1. Expanding Access to Genomic Medicine

- Essential to ensure equal access to genomic medicine for patients with poor transportation options.
- Online is especially suitable for services that do not require repeated visits.
- Reduces physical, mental, and financial burden for patients traveling long distances.
- Online implementation may help reach patients who have not yet received CGP testing.

2. Clinical Appropriateness and Continuity

- Online consultations were already used when patients were unwell, without any sense of mismatch.
- Online CGP explanations worked well during the COVID-19 pandemic.
- Explanations and follow-up care are nearly identical whether delivered online or in person.
- Emotional cues such as facial expressions and tone of voice can still be read in online settings.

3. Ensuring Equity and Regional Needs

- Online systems should be allowed to support fairness and equal access to care.
- In non-urban areas, few physicians are capable of handling CGP; online care is therefore necessary.
- CGP access is becoming a bottleneck to downstream treatments like clinical trials.

4. Patient-Centered Efficiency

- Young patients eager to participate in trials may benefit from direct, centralized access.
- Online care may enable faster engagement with core institutions and trial sites.

Table S2 Barriers and Considerations for Online Implementation of CGP

1. Patient Eligibility and Comprehension
   - Assessing eligibility (e.g., ECOG PS) is difficult without face-to-face interaction.
   - Understanding of CGP differs among patients and referring physicians.
   - Referring physicians from non-genomic hospitals need a basic understanding of CGP.
   - Referring physicians may need to assist patients in understanding results.
   - Face-to-face settings are easier for elderly patients to ask questions and communicate.
   - Many elderly patients struggle with digital tools.
2. Information Delivery and Explanation Method
   - In-person explanation is essential for hereditary disease results; genetic counselors should attend in person.
   - Separating result disclosure and genetic counseling may reduce counseling completion.
   - The treating physician should be present for result disclosure as it affects treatment decisions.
   - Online consent may be unsuitable due to the volume of information to explain.
   - Online consent may not be suitable for tests like tumor-normal pairs or liquid biopsies.
3. Technical and Institutional Challenges
   - Institutional setup is required, making implementation difficult.
   - Provides little benefit to the institution and increases burden.
   - Online explanation is less efficient and does not reduce workload.
4. Security and Trust
   - Confidentiality concerns exist when handling genetic data online.
   - Concerns about possible recording of online sessions.
   - Risk of complications or disputes exists.
   - Payment disputes may arise if results are disappointing.
5. Coordination and Communication Needs
   - Aligning understanding between all parties requires time and effort.
   - Prefer patients visit our facility while referring physicians join online.
6. Feasibility and Current Practices
   - Most referrals are from nearby hospitals, so the need for online-only explanation has decreased.
   - A few in-person visits (e.g., two) are acceptable.
